# Simulated Microgravity Influences VEGF, MAPK, and PAM Signaling in Prostate Cancer Cells

**DOI:** 10.3390/ijms21041263

**Published:** 2020-02-13

**Authors:** Trine Engelbrecht Hybel, Dorothea Dietrichs, Jayashree Sahana, Thomas J. Corydon, Mohamed Z. Nassef, Markus Wehland, Marcus Krüger, Nils E. Magnusson, Johann Bauer, Kirsten Utpatel, Manfred Infanger, Daniela Grimm, Sascha Kopp

**Affiliations:** 1 Department of Biomedicine, Aarhus University, 8000 Aarhus C, Denmarkjaysaha@biomed.au.dk (J.S.); corydon@biomed.au.dk (T.J.C.); 2 Clinic for Plastic, Aesthetic and Hand Surgery, Otto von Guericke University Magdeburg, 39120 Magdeburg, Germany; dorothea.dietrichs@st.ovgu.de (D.D.); mohamed.nassef@med.ovgu.de (M.Z.N.); markus.wehland@med.ovgu.de (M.W.); marcus.krueger@med.ovgu.de (M.K.); manfred.infanger@med.ovgu.de (M.I.); 3 Department of Ophthalmology, Aarhus University Hospital, 8200 Aarhus N, Denmark; 4 Research Group “Magdeburger Arbeitsgemeinschaft für Forschung unter Raumfahrt-und Schwerelosigkeitsbedingungen” (MARS), Otto von Guericke University, Universitätsplatz 2, 39106 Magdeburg, Germany; 5 Diabetes and Hormone Diseases—Medical Research Laboratory, Department of Clinical Medicine, Faculty of Health, Aarhus University, 8200 Aarhus N, Denmark; nm@clin.au.dk; 6 Max Planck Institute of Biochemistry, 82152 Martinsried, Germany; jbauer@biochem.mpg.de; 7 Institute for Pathology, University of Regensburg, 95053 Regensburg, Germany; kirsten.utpatel@ukr.de; 8 Department of Microgravity and Translational Regenerative Medicine, Otto von Guericke University, Pfälzer Platz, 39106 Magdeburg, Germany

**Keywords:** microgravity, prostate cancer, VEGF signaling, cytoskeleton, focal adhesion, extracellular matrix

## Abstract

Prostate cancer is one of the leading causes of cancer mortality in men worldwide. An unusual but unique environment for studying tumor cell processes is provided by microgravity, either in space or simulated by ground-based devices like a random positioning machine (RPM). In this study, prostate adenocarcinoma-derived PC-3 cells were cultivated on an RPM for time periods of 3 and 5 days. We investigated the genes associated with the cytoskeleton, focal adhesions, extracellular matrix, growth, survival, angiogenesis, and metastasis. The gene expression of signaling factors of the vascular endothelial growth factor (VEGF), mitogen-activated protein kinase (MAPK), and PI3K/AKT/mTOR (PAM) pathways was investigated using qPCR. We performed immunofluorescence to study the cytoskeleton, histological staining to examine the morphology, and a time-resolved immunofluorometric assay to analyze the cell culture supernatants. When PC-3 cells were exposed to simulated microgravity (s-µg), some cells remained growing as adherent cells (AD), while most cells detached from the cell culture flask bottom and formed multicellular spheroids (MCS). After 3-day RPM exposure, PC-3 cells revealed significant downregulation of the *VEGF*, *SRC1*, *AKT*, *MTOR*, and *COL1A1* gene expression in MCS, whereas *FLT1*, *RAF1*, *MEK1*, *ERK1*, *FAK1*, *RICTOR*, *ACTB*, *TUBB*, and *TLN1* mRNAs were not significantly changed. *ERK2* and *TLN1* were elevated in AD, and *FLK1*, *LAMA3*, *COL4A5*, *FN1*, *VCL*, *CDH1*, and *NGAL* mRNAs were significantly upregulated in AD and MCS after 3 days. After a 5-day culture in s-µg, the PC-3 cells showed significant downregulations of *VEGF* mRNA in AD and MCS, and *FN1*, *CDH1*, and *LAMA3* in AD and *SCR1* in MCS. In addition, we measured significant upregulations in *FLT1*, *AKT*, *ERK1*, *ERK2*, *LCN2*, *COL1A1*, *TUBB*, and *VCL* mRNAs in AD and MCS, and increases in *FLK1*, *FN1*, and *COL4A5* in MCS as well as *LAMB2*, *CDH1*, *RAF1*, *MEK1*, *SRC1*, and *MTOR* mRNAs in AD. *FAK1* and *RICTOR* were not altered by s-µg. In parallel, the secretion rate of VEGFA and NGAL proteins decreased. Cytoskeletal alterations (F-actin) were visible, as well as a deposition of collagen in the MCS. In conclusion, RPM-exposure of PC-3 cells induced changes in their morphology, cytoskeleton, and extracellular matrix protein synthesis, as well as in their focal adhesion complex and growth behavior. The significant upregulation of genes belonging to the PAM pathway indicated their involvement in the cellular changes occurring in microgravity.

## 1. Introduction

Space research and investigations on cell signaling processes in the growth and development of cells exposed to microgravity (µg) is currently a hot topic in cancer research and regenerative medicine [1,2,3,4,5]. Prostate cancer is the fifth leading cause of cancer mortality in men, with the second highest cancer incidence worldwide. It is the most frequently diagnosed cancer in more than half of the countries in the world. In 2018, 1,276,106 cases of prostate cancer and 358,989 deaths were recorded worldwide [6]. Therefore, it is important to increase the current knowledge and develop novel treatment strategies for prostate cancer.

According to the 2016 World Health Organization (WHO) classification of tumors of the prostate, one of the main groups of prostate carcinomas are epithelial tumors, including the subtype acinar adenocarcinoma [7], which is by far the most common type of prostate carcinoma [8,9]. Initially, this carcinoma is local and confined to the prostate. If not detected early, it can progress to advanced stages, characterized by local invasion of the vesicular glands. The cancer can eventually progress to metastatic disease, usually resulting in death [9]. Moreover, the disease can change to a state of androgen independence, increasing the difficulty of treatment [9].

Many studies have shown that changes in gravity have extensive effects on cell functions [10,11,12]. Microgravity can induce alterations in morphology, growth, and protein biosynthesis and secretion [10], depending on the responding cell type [12]. In addition, cell cultures in µg can be performed scaffold-free to engineer three-dimensional (3D) aggregates, including multicellular spheroids (MCS), tissues, and tubular structures [13,14,15]. These organoids can be used for research in space medicine [13,16] and for testing the delivery and efficacy of drugs [17].

However, real µg can only be experienced during spaceflights, unmanned Bion flights, suborbital flights, rocket flights, parabolic flights, or with the help of a drop tower [18]. As these opportunities are rare and costly, devices have been developed to simulate µg on Earth, including the random positioning machine (RPM), rotating wall vessel (RWV), and the two-dimensional (2D) or 3D clinostat [16,18].

An RPM consists of two independently rotating frames, each moving at a randomized speed and direction [16]. By placing a sample of sufficiently small objects or cells in the middle of the RPM, the gravity vector perceived by the sample is averaged to zero over time; thus, the objects are exposed to simulated µg (s-µg) [16,19]. It has been shown for various cell types, including thyroid [20] and breast cancer cells [21], that cultivation on an RPM results in MCS formation [16].

This method facilitates long-term scaffold-free 3D-aggregate formation with the advantage of a lack of necrosis. This makes it possible to conduct long-term experiments where huge spheroids can be produced [22]. Experiments with follicular thyroid cancer cells cultured in a scaffold-free manner on an RPM for 14 days [23] and endothelial cells exposed to s-µg for 28 and 35 days revealed no necrosis. The endothelial cells grew in a large number of 3D organoids and tubular structures [15,24]. The liquid overlay and spinner flask techniques and other 1 g methods provide specific environments for 3D formation [25]. Unfortunately, after two weeks the spheroids revealed a necrotic center [26].

MCS are spherical cell clusters containing networks of cell–cell and cell–matrix interactions [17]. They are similar to in vivo tissue regarding the complexity of cell types, cell–cell interactions, extracellular matrix (ECM) deposition, and chemical gradients [17]. The concentration of oxygen and nutrients is highest in the outer layers, while metabolic wastes accumulate in the center [17,27], which resembles the conditions in avascular tumors but is “inside-out” compared to tumors containing capillaries in the center [28]. It has been shown that when MCS are formed from cancer cells, they mimic tumors formed in vivo [29] better than monolayers of cells [28]. Therefore, they can be used as in vitro model systems and for high-throughput screening assays [30] to measure the responses of tumors to potential anticancer drugs [28,29,30]. This increases the opportunity for cell-based research in drug development [30] and can be used as an addition to in vivo models and/or to reduce animal experiments [28].

Various studies have shown that µg can affect the secretion of vascular endothelial growth factors (VEGFs) in different cell types [15,23,31]. VEGFs are signaling molecules important for the process of angiogenesis [32]. Abnormal angiogenesis is one of the hallmarks of cancer [33], and overexpression of VEGF-A has been implicated in pathological angiogenesis in tumors and other diseases, causing the development of dysfunctional and disorganized types of blood vessels [32]. In tumors, neoangiogenesis changes blood flow, oxygenation, and interstitial fluid pressure, creating an abnormal microenvironment, increasing cancer progression, and decreasing the efficacy of treatment [33]. Anti-VEGF treatment has been developed and successfully utilized as cancer treatment [33,34].

The actions of VEGF-A are mediated through the molecule binding to its two receptors, VEGFR-1/fetal liver kinase 1 (FLT1) and VEGFR-2 Fms-related tyrosine kinase 1 (FLK1). Upon VEGF-A binding to VEGFR-2, phosphorylation of specific tyrosine residues leads to recruitment of downstream effector proteins and initiation of specific signaling pathways [35]. The extracellular signal regulated kinase 1/2 (ERK1/2)/rapidly accelerated fibrosarcoma (raf)-mitogen-activated protein kinase kinase 1 (MEK1)-mitogen-activated protein kinase (MAPK) pathway is activated, leading to increased proliferation [35,36]. Apoptosis is inhibited through the steroid receptor coactivator (SRC) proto-oncogene, non-receptor tyrosine kinase (Src)/phosphoinositide 3-kinase (PI3K)/protein kinase B (PKB, also known as Akt) signaling pathway, leading to increased cell survival [35,37]. Mammalian target of rapamycin (mTOR) signaling, which includes mTOR complex 2 (mTORC2), containing the subunits mTOR and rapamycin-insensitive companion of mTOR (RICTOR), is activated through the PI3K/Akt signaling pathway, regulating processes such as cell growth and differentiation [38]. VEGF signaling through VEGFR-2 also leads to activation of focal adhesion kinase (FAK), which stimulates increased vascular permeability [39] as well as migration and anti-apoptosis via Src [40]. Moreover, p38MAPK is activated, affecting cell motility through actin cytoskeleton reorganization [35].

In comparison to other VEGFRs, the signaling functions of VEGFR-1 are less well characterized [41]. It has been associated with the ERK/MAPK, PI3K/AKT, and p38MAPK signaling pathways [42]. Furthermore, VEGFR-1 has been shown to bind VEGF with a higher affinity than VEGFR-2 and thereby have a negative impact on angiogenesis caused by VEGFR-2 [41].

Previous findings showed 3D growth patterns of PC-3 cells when cultured in an RWV culture system [14]. Spheroid formation followed within a few hours after reducing gravitational conditions, and expression of cell adhesion molecules (CD44 and E-cadherin) was increased in the MCS. In parallel, Zhau and co-workers established a 3D human prostatic cancer model by co-culturing prostate fibroblasts with prostate cancer cells under s-µg conditions using an RWV system [43]. Using a similar setup, Clejan and colleagues studied the effects of RWV cultivation on DU-145 human prostate carcinoma cells [44]. Notably, after 6 days of RWV growth, the activation of the PI3K pathway was observed. From this study the authors concluded that RWV cultivation provides a 3D growth model of prostate cancer that imitates in vivo tissue growth. In addition, Margolis et al. reported long-term maintenance (minimum of 28 days) of benign explanted human prostate tissue grown in culture medium in an RWV [45]. This study investigated the impact of s-µg on PC-3 prostate cancer cells in vitro.

The principal aims of this study are to (i) investigate the changes and behavior of PC-3 prostate cancer cells exposed to s-µg created by an RPM; (ii) test, based on previous findings, the hypothesis that exposure of PC-3 cells to s-µg induces the formation of MCS and alters the expression of genes related to angiogenesis, metastasis, the cytoskeleton, the ECM, and focal adhesions (FAs); and (iii) focus on the VEGF, MAPK, and PI3K/AKT/mTOR (PAM) signaling pathways.

## 2. Results

### 2.1. Morphology and Cell Growth

In order to examine whether PC-3 cells will form MCS when cultured under s-µg conditions, cell culture flasks containing PC-3 cells were placed on the RPM for 3 and 5 days; 1 g controls were cultured in parallel.

**Figure 1:**
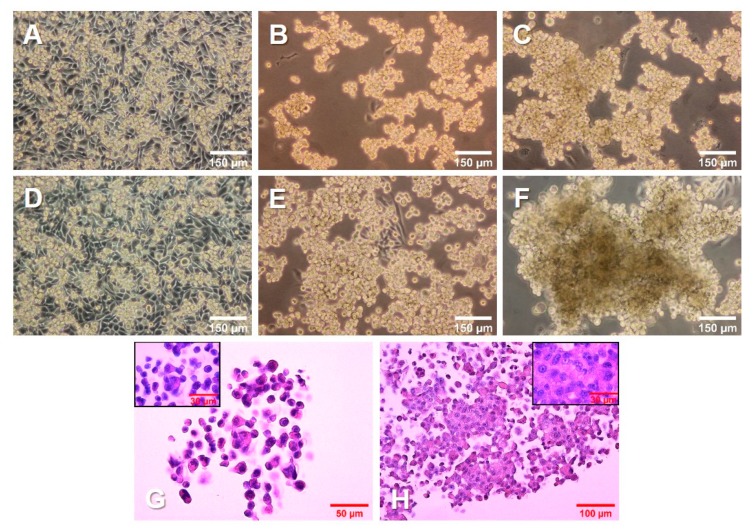
Growth of PC-3 prostate cancer cells in simulated microgravity. PC-3 cells were cultured (**A**, **D**) under 1 g conditions or (**B**,**C**,**E**,**F**) on an RPM for 3 days (**A**–**C**) and 5 days (**D**–**F**). (**G**,**H**) PC-3 cells growing in MCS, stained with hematoxylin and eosin (HE) after a 3-day (**G**) and 5-day (**H**) RPM exposure, respectively.

The 1 g control cells (Figure 1A,D) kept dividing throughout the experiment, reaching 100% confluence. The cells on the RPM (Figure 1B,C,E,F) were exposed to s-µg conditions. Only a few cells remained adherent, growing as a 2D monolayer (AD), while the majority detached and aggregated to MCS. After 3 days (Figure 1B,C), there were numerous MCS floating in the medium. In addition, a smaller number of loose aggregates could be found among the spheroids. After 5 days (Figure 1E,F), the spheroids had grown significantly. The size, shape, and density varied among the spheroids, as well as among the cell culture flasks used in the same experiment.

Hematoxylin–eosin (HE) staining of spheroids grown after 3 days (Figure 1G) and 5 days (Figure 1H) on the RPM presented a condensation of cells together with an increased amount of ECM. In addition, the cells in all groups were viable and did not undergo apoptosis or necrosis, as seen from the normal cell shape, cytoplasm staining, and intact nuclei.

### 2.2. VEGF, MAPK, and PAM Signaling Pathways

**Figure 2:**
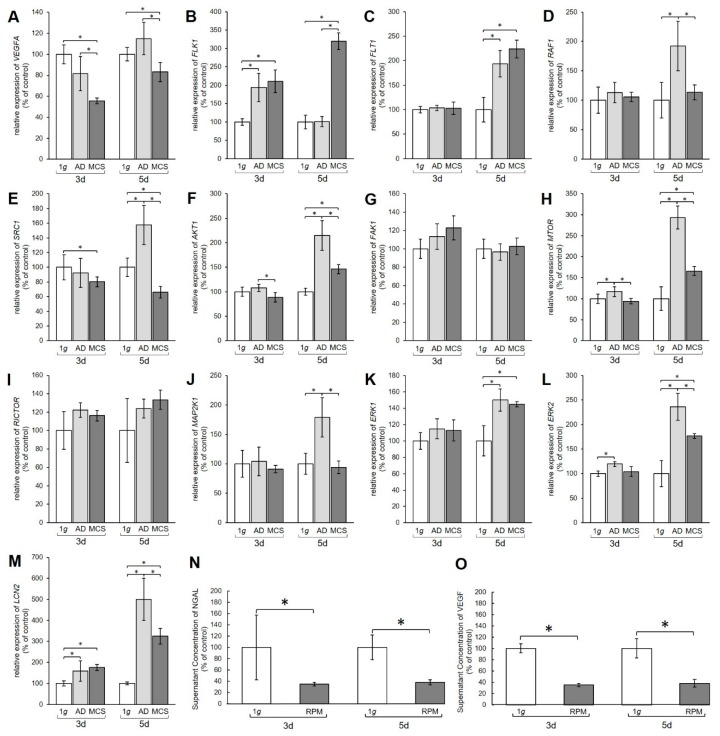
Gene expression of various proteins from the vascular endothelial growth factor (VEGF) signaling pathway. Results show relative mRNA transcription levels from (**A**) *VEGFA*, (**B**) *FLK1*, (**C**) *FLT1*, (**D**) *RAF1*, (**E**) *SRC1*, (**F**) *AKT1*, (**G**) *FAK1*, (**H**) *MTOR*, (**I**) *RICTOR*, (**J**) *MAP2K1*, (**K**) *ERK1*, (**L**) *ERK2*, and *(***M***) LCN2* genes. Results are shown as percentages of 3-day and 5-day 1 g controls. Time-resolved immunofluorometric assay (TRIFMA) results show amounts of (**N**) NGAL and (**O**) VEGF proteins in the supernatant taken from 3-day and 5-day flasks containing PC-3 cells, shown as percentages of 3-day and 5-day 1 g controls. * *p* < 0.05.

Quantitative real-time polymerase chain reaction (qPCR) analysis was performed to examine the gene expression of relevant genes of the VEGF, MAPK, and PAM signaling pathways. The qPCR data revealed a significant decrease in the amount of *VEGFA* mRNA in MCS compared to 1 g controls after 3 days and 5 days (Figure 2A).

The mRNA levels of the VEGFA receptors *FLK1* and *FLT* also showed changes (Figure 2B,C). After 3 days the *FLK1* gene expression was significantly upregulated in both AD and MCS compared to 1 g. After 5 days the *FLK1* mRNA level in AD was unchanged, while MCS showed an increase compared to 1 g and AD (Figure 2B). The *FLT1* mRNA was unchanged after 3 days, but clearly upregulated in both 5-day AD and MCS groups compared with 1 g controls (Figure 2C).

Furthermore, the MAPK pathway was affected by the s-µg conditions. The *RAF1* mRNA was not changed after 3 days, but significantly increased in AD cells after 5 days (Figure 2D). The *SRC1* mRNA expression was significantly downregulated in 3-day MCS compared to corresponding 1 g samples, whereas a 5-day exposure induced upregulation of *SRC1* mRNA in AD and downregulation in MCS compared with 1 g controls and AD samples (Figure 2E). *AKT1* was downregulated in MCS compared to AD, but not significantly changed compared with 1 g after a 3-day RPM exposure. After 5 days, we measured an upregulation of the *AKT1* gene in AD and MCS compared with 1 g (Figure 2F). Interestingly, the *FAK1* gene expression was not significantly changed in all samples (Figure 2G). *MTOR* mRNA was significantly elevated in AD after 3 days and further enhanced after 5 days in both AD and MCS samples (Figure 2H). *RICTOR* gene expression was not differentially displayed in all groups (Figure 2I). Similarly, there was also no change in *MAP2K1* mRNA after 3 days, whereas after 5 days *MAP2K1* mRNA was significantly upregulated in AD cells (Figure 2J). *ERK1* was not changed in 3-day samples, but significantly enhanced in AD and MCS after 5 days (Figure 2K). *ERK2* was upregulated in AD samples after 3 days and, like *ERK1*, clearly upregulated in AD and MCS after 5 days (Figure 2L).

The transcription level of the lipocalin 2/NGAL protein neutrophil gelatinase-associated lipocalin (*LCN2*) gene, however, was increased in both AD and MCS compared to 1 g after 3 and 5 days, but decreased from AD to MCS after 5 days (Figure 2M). Furthermore, the amount of VEGF and NGAL protein secreted from the PC-3 cells into the medium was examined using time-resolved immunofluorometric assay (TRIFMA) (Figure 2N,O). Secreted amounts of VEGF and NGAL were significantly decreased after both 3 and 5 days of RPM-exposure.

### 2.3. Cytoskeleton

**Figure 3:**
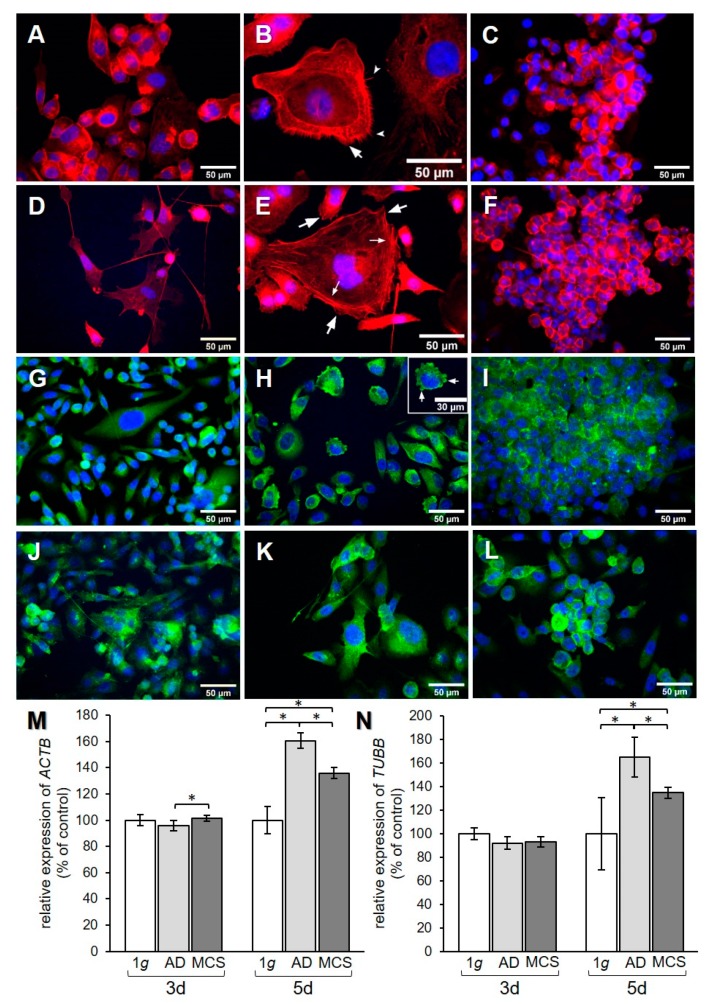
Investigation of the cytoskeleton. F-actin visualized by means of phalloidin and immunofluorescence (IF) staining for β-actin. F-actin pictures show (**A**) 3-day 1 g control, (**B**) 3-day AD, (**C**) 3-day MCS, (**D**) 5-day 1 g, (**E**) 5-day AD, and (**F**) 5-day MCS. Big arrows in (**B**) and (**E**) point to lamellipodia, notched arrows in (**B**) point to filopodia, and small arrows in (**E**) point to stress fibers. β-actin pictures show (**G**) 3-day 1 g control, (**H**) 3-day AD, (**I**) 3-day MCS, (**J**) 5-day 1 g, (**K**) 5-day AD, and (**L**) 5-day MCS. Arrows in (**H**) point to membrane blebbing. Relative mRNA transcription levels of (**M**) *ACTB* and (**N**) *TUBB* genes in 3-day and 5-day samples were analyzed by qPCR. * *p* < 0.05.

The effect of RPM exposure on the cytoskeleton was examined by qPCR analysis for the genes encoding β-actin and β-tubulin. In addition, F-actin and β-actin were visualized by immunofluorescence (IF). A redistribution of F-actin was observed in PC-3 cells when exposed to s-µg for 3 and 5 days (Figure 3A–C and Figure 3D–F, respectively). AD cells showed an accumulation of cortical actin compared to 1 g control cells. After 3 days, the formation of filopodia and lamellipodia could be observed (Figure 3B). After 5 days, filopodia, lamellipodia, and cortical stress fibers were prominent (Figure 3E). F-actin was accumulated at the cell membrane in MCS after both 3 and 5 days (Figure 3C,F). IF staining for β-actin (Figure 3G–L) did not show a clear difference in the distribution of the protein, although the AD at 3 days (Figure 3H) showed some membrane blebbing. *ACTB* gene expression revealed a significant upregulation in MCS compared to AD after 3 days (Figure 3M), while *TUBB* presented no expression changes (Figure 3N). After 5 days, *ACTB* and *TUBB* mRNAs were increased in both AD and MCS compared to 1 g control cells, and were lower in MCS than AD (Figure 3M,N).

### 2.4. Extracellular Matrix

**Figure 4:**
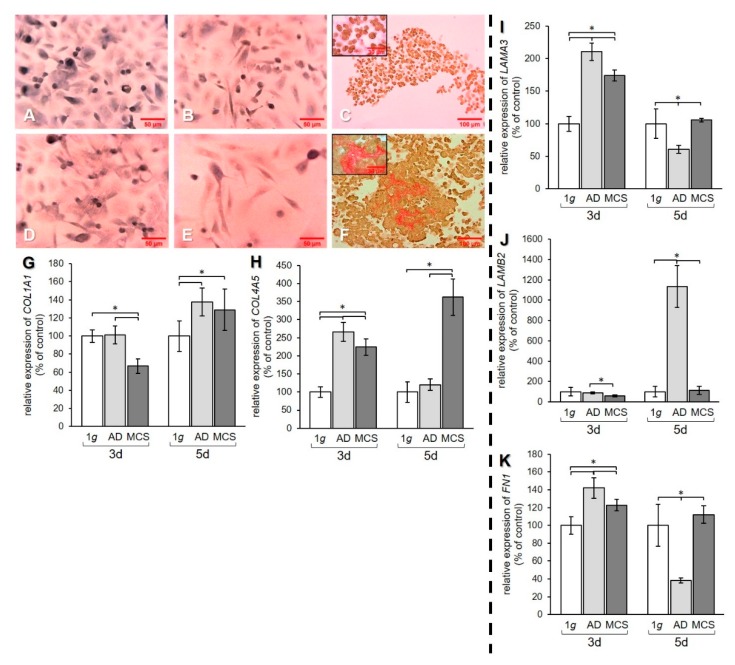
Investigation of ECM components. Collagen deposition in PC-3 cells stained with Sirius red (SR) after (**A**–**C**) 3 days and (**D**–**F**) 5 days. 1 g control cells (**A**,**D**) AD (**B**,**E**) and MCS (**C**,**F**). Depositions of collagen I and III are stained in red. qPCR results show relative mRNA transcription levels of (**G**) *COL1A1*, (**H**) *COL4A5*, (**I**) *LAMA3*, (**J**) *LAMB2*, and (**K**) *FN1* genes in 3-day and 5-day AD and MCS. * *p* < 0.05.

To examine the effect of s-µg on the ECM, the regulation of collagen, laminin, and fibronectin at the mRNA level and collagen deposition were investigated (Figure 4).

Sirius red staining of PC-3 cells revealed that 1 g cell samples (Figure 4A,D) and AD (Figure 4B,E) were negative for extracellular collagen after 3 and 5 days. However, collagen was found in RPM-exposed samples and was detectable in MCS. After 3-day exposure, a rim of collagen was formed around the cells in the MCS (Figure 4C). After 5 days, larger amounts of collagen were detectable in the center of the MCS, as demonstrated by the prominent red structures (Figure 4F).

qPCR data of the *COL1A1* gene expression revealed a decrease of this gene in MCS compared to both 1 g and AD after 3 days. After 5 days, an upregulation of *COL1A1* mRNA was measured in both AD and MCS compared to 1 g controls (Figure 4G). The gene expression of *COL4A5* was increased after 3 days in AD and MCS compared to 1 g, but decreased in MCS compared to AD. After 5 days, the *COL4A5* mRNA level was strongly elevated in MCS, while no difference was found between AD and 1 g (Figure 4H). The mRNA expression of the genes for the laminin α3 and β2 subunits was investigated. The mRNA level of the *LAMA3* gene was upregulated in AD and MCS compared to 1 g after 3 days, while the *LAMA3* mRNA level in MCS was lower than in AD. After 5 days, the *LAMA3* mRNA in AD was decreased compared to both 1 g and MCS, while MCS did not show a difference compared to 1 g (Figure 4I).

The *LAMB2* mRNA level in AD and MCS was unchanged after 3 days compared to 1 g. *LAMB2* was downregulated in MCS compared to AD. After 5 days, the *LAMB2* mRNA transcription was very high in AD compared to both 1 g and MCS (Figure 4J). The qPCR data for *FN1* showed an increase in AD and MCS cells compared to 1 g after 3 days (Figure 4K). After 5 days, the *FN1* mRNA level was decreased in AD samples compared to both 1 g and MCS (Figure 4K).

### 2.5. Altered Expression of Genes of the Focal Adhesion Complex

Finally, we examined the effect of s-µg-exposure of PC-3 cells on the transcription of genes involved in FA.

**Figure 5:**
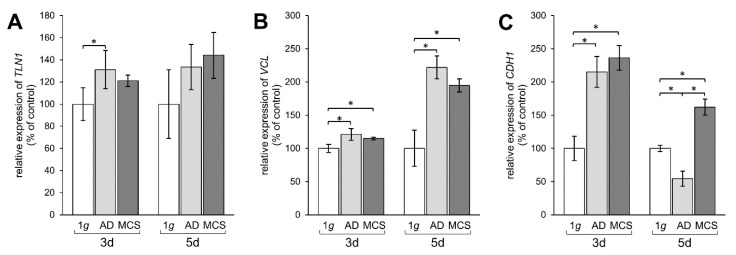
Investigation of gene expression of focal adhesion molecules talin-1, vinculin, and E-cadherin. qPCR results show relative mRNA transcription levels of (**A**) *TLN1*, (**B**) *VCL*, and (**C**) *CDH1* genes in 3-day and 5-day samples. * *p* < 0.05.

*TLN1* gene expression was upregulated mRNA level after 3 days in AD, but not in MCS compared to 1 g, whereas after 5 days no significant changes were measured (Figure 5A). The mRNA expression of *VCL* was significantly elevated in AD and MCS samples compared to 1 g controls. This elevation of *VCL* was further enhanced after 5 days in both AD and MCS (Figure 5B). Moreover, the *CDH1* gene expression was clearly upregulated after an RPM-exposure in 3-day AD and MCS samples. In contrast, the *CDH1* mRNA expression was downregulated in AD after 5 days, while the *CDH1* level was still upregulated in MCS compared to both 1 g and AD (Figure 5C).

### 2.6. Interaction of Genes Investigated by Pathway Analysis

**Figure 6:**
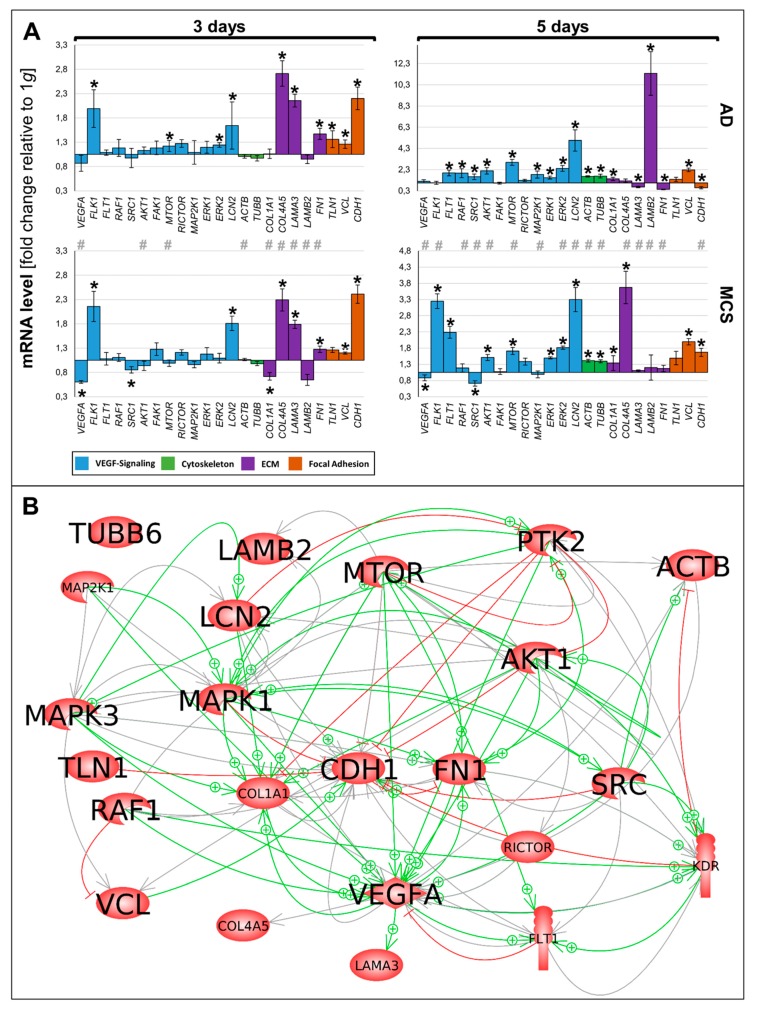
(**A**) Summary of gene expression fold change measured by qPCR of 3- and 5-day RPM-exposed PC-3 samples. Results determined in AD and MCS in relation to 1 g are shown. Color-coded bars represent genes of related biological processes. * *p* < 0.01 1 g vs. AD/MCS, # *p* < 0.01 AD vs. MCS. (**B**) Interaction network of selected items at gene expression level; 22 of 23 selected genes analyzed by qPCR contribute to the network comprising 114 relations. Green arrows indicate activation and red arrows indicate inhibition. Gray lines signify that interactions take place whose effects have not been clarified. The interaction network was built using Elsevier Pathway Studio plus.

The various genes analyzed by qPCR (Figure 6A) were differentially regulated in µg samples (AD and RPM). Figure 6A presents a summary of the qPCR data, already described in Figure 2, Figure 3, Figure 4 and Figure 5, and gives a comparable overview on the results. A closer look at the 3-day samples reveals that most genes involved in VEGF signaling (blue bars) are upregulated in AD samples compared to 1 g, while the gene expression in MCS presents a more heterogeneous pattern. Notably, *VEGFA*, *FLK1*, and *LCN2* are equally regulated in AD and MCS samples after 3 days. After a 5-day RPM-exposure, most of the investigated VEGF signaling molecules are significantly upregulated in AD samples. In comparison, 5-day MCS samples presented a significant regulation, which is in contrast to 3-day samples. Remarkably, *VEGFA* is not regulated in 5-day AD samples compared to 1 g. In addition, *LCN2* is strongly upregulated in all conditions. The cytoskeletal molecules of interest (green bars) are not regulated after 3 days. However, after 5 days, both conditions are upregulated. The investigated ECM molecules (violet bars) after a 3-day RPM-exposure present a comparable image in AD and MCS samples. After 5 days, AD cells show a heterogeneous expression image, while *LAMB2* is strikingly upregulated. In MCS, however, collagens are clearly enhanced, while *LAMB2* is not changed compared to the controls. Genes of the focal adhesion molecule complex (orange bars), especially *CDH1* mRNA, are highly upregulated in both 3-day conditions. Even though *CDH1* is still significantly upregulated, it seems to be reduced in 5-day conditions.

The 23 genes of interest were investigated in regard to their 114 possible interaction and mutual expression dependence. A pathway analysis of these items, represented in molecular action mode, is shown in Figure 6B. It can be seen that the components investigated are members of a complicated network, which includes the central factors *CDH1*, *VCL*, *TLN1*, *FN1*, *VEGFA*, *KDR*, *RAF1*, *ERK1/2*, *MAP2K1*, *MTOR*, *AKT1*, *PTK2*, and *SRC1*. Of these components, *CDH1* seemed to be the main node point.

## 3. Discussion

In this study, we focused on the changes induced in PC-3 cells when they were cultured under s-µg conditions. Possible simulation devices included RPM, RVW, and 2D clinostat. The RPM was chosen as this device rotates samples around two axes, thus enabling the cells to float freely and randomly in the medium, which increases the chances of interactions and MCS formation [16]. In addition, the RPM provides efficient diffusion of nutrients, gases, and waste through a small fluid flow [16]. The optimal method would be to cultivate the cells on a spaceflight, as this would provide real µg. In contrast, simulation devices average the gravity level to near zero without neutralizing it [18] and expose the cells to small fluid shear forces [46], which might slightly decrease the reproducibility in real µg. However, previous studies have noted that in many cases s-µg produces results similar to real µg [47]. Genes and proteins involved in the regulation of cancer cell proliferation and metastasis, such as IL6, IL8, and VEGFA, were similarly regulated under RPM and spaceflight conditions [47]. Therefore, in the future, it would be interesting to expose PC-3 cells to real µg to compare its effects to our results, as only space experiments can validate results obtained on devices on Earth [18].

When PC-3 cells were placed on the RPM, some of the cells remained adherent, while others detached and formed multicellular tumor-like spheroids. After 5 days, only a few cells remained growing adherently, while the majority were growing in MCS. Notably, variations in the shape, size, and density of the spheroids were observed. This could be due to variations in placement on the RPM. Nevertheless, various cell types exposed to s-µg have grown as multicellular spheroids and also as an adherent monolayer. Examples are thyroid cancer cells [20], breast cancer cells [21], and nonmalignant cells, such as endothelial cells [15].

### 3.1. Signaling Pathways Involved in Three-Dimensional Growth

#### 3.1.1. VEGF Signaling

We examined the expression of genes belonging to the VEGF signaling pathway and found that the gene expression of *VEGFA* was downregulated after 3 and 5 days of exposure, except in 5-day AD. Accordingly, significant decreases in the amount of secreted VEGF and NGAL were found. This points toward development of a less-aggressive phenotype as a result of cultivation on the RPM, as VEGFA has been implicated in pathological angiogenesis and tumor development [32,48], while NGAL induces *VEGFA* expression [49], among other cancer-promoting functions [50,51,52]. The *LCN2* gene expression, on the other hand, was increased in all groups, potentially due to a counterregulatory mechanism. Possibly, mRNA levels increase in order to compensate the elevated protein secretion of NGAL protein in the supernatant. The development of a less-aggressive phenotype is supported by the findings in thyroid cancer cells [22,47] and human adult retinal pigment epithelium cells [31]. In contrast, the VEGF and NGAL secretion was elevated in endothelial cells exposed to µg [15]. Future studies should explore this µg-induced shift in greater detail. This can be elucidated by investigating the regulation of other genes and proteins linked to metastasis and proliferation of cancer cells, such as interleukin 6 (IL6), IL8, osteopontin, and fibroblast growth factor 17 [22,47].

In contrast to these findings, we found the expression of genes for the VEGF receptors *FLT1* and *FLK1* to be upregulated after 5 days in MCS, which might indicate a counterregulatory effect of the two VEGF receptors. Furthermore, we found a significant regulation of downstream signaling molecules, with a general trend to upregulate the gene expression. This was especially evident after 5 days on the RPM.

#### 3.1.2. MAPK Signaling

Factors of the MAPK signaling pathway are known to be involved in 3D growth and metastasis [1]. The biological process of the epithelial–mesenchymal transition (EMT) is known to increase migration and spreading of cancer cells, progression of the cell cycle, and resistance to apoptosis and chemotherapy [53]. Thus, we focused on *RAF1* mRNA expression. RAF1 acts as a regulatory link between the membrane-associated Ras GTPases and the MAPK/ERK cascade and functions as a switch determining, among others, proliferation, differentiation, and oncogenic transformation of human cells. Its main cellular role is in phosphorylation and activation of the MAP kinase kinases MEK1 and MEK2 [54].

In this study we found *RAF1* to be upregulated in AD cells but not in MCS. This indicates its activation by s-µg and its possible involvement in spheroid formation (Figure 2D). As RAF1 activation initiates a mitogen-activated protein kinase cascade, we also determined *MAP2K1* (also known as *MEK1*) gene expression. *MAP2K1* gene expression was comparable to the *RAF1* mRNA level and was significantly elevated in 5-day AD cells (Figure 2J). In a next step, we determined the expression of *ERK1* and *ERK2.* The ERK subfamily consists of typical (ERK 1/2/5) and atypical (ERK 3/4/7/8) members. ERKs are known to regulate EMT and promote tumor progression [54]. *ERK1/2* gene expression was significantly upregulated after 5 days in AD and MCS compared to 1 g samples (Figure 2K,L). Both ERK1 and ERK2 seem to be involved in 3D aggregation in µg.

#### 3.1.3. PI3K/AKT/mTOR (PAM) Signaling

Tee et al. showed that the PAM signaling pathway is frequently mutated in prostate cancer [55]. The PAM pathway is involved in the regulation of growth, metabolism, and migration [55]. In our experiment, the *AKT* gene expression was enhanced in AD and MCS samples after a 5-day RPM-exposure of prostate cancer cells. Activated AKT phosphorylates a host of proteins involved in cell growth, including mTOR [56]. Interestingly, we observed an upregulation of the *MTOR* gene expression in AD after 3 days and in all RPM-exposed cell samples after 5 days. According to the clinicaltrials.gov database, various inhibitors of this pathway are currently being tested in 36 phase I/II clinical trials for prostate cancer, either as monotherapy or in combination with conventional chemotherapies [57].

Finally, we focused on the *SRC1* gene, which belongs to the p160 family of SRCs. SRCs promote cancer cell proliferation, survival, metabolism, migration, invasion, and metastasis. In prostate cancer, *SRC1* is highly expressed [58]. We found an upregulated *SRC1* gene in AD cells after 5 days, indicating its involvement in migration and 3D formation.

Together with MAP1K2, SRC-1 regulates the transcriptional activity of STAT3 [59]. In addition, it blocks upregulation of the VEGF-A receptors [60] and inhibits expression of E-cadherin [61]. On the other side, SRC-1 activates FAK1 [62], which strengthens vinculin, containing focal adhesion complexes [63] that link cells to fibronectin or other components of the ECM [64] and include talin-1 as a hinge between the cytoskeleton and the extracellular space [65]. Downregulation of talin favors activation of the MAPK1/MAPK3 pathway [66].

### 3.2. Changes in the Cytoskeleton and the Extracellular Matrix

In this study we observed s-µg-induced changes in the F-actin network of the cells. The formation of lamellipodia and filopodia in our cells under s-µg might indicate that the cells are trying to adhere better to the ECM to resist the s-µg conditions, because these structures are involved in migration and adherence of cells to the substrate [67]. In addition, the stress fibers also present might have formed in order to compensate for changes in adhesion and cell morphology [68].

The cortical F-actin accumulation is consistent with earlier findings in endothelial cells, although the latter also showed perinuclear accumulation [12]. An increase in the density of F-actin was also found in A431 cells, although the actin filaments became less organized [69]. Just like PC-3 cells, human fetal osteoblasts showed formation of filopodia and lamellipodia [70]. In contrast, osteoblasts had reduced cortical actin and fewer stress fibers [71]. It is clear from these examples that the changes in F-actin induced by µg depend on the cell type.

Moreover, we found that the mRNA level of *ACTB* was increased after 5 days. This is in accordance with similar findings in follicular thyroid cancer cells [72]. In addition, our qPCR analysis showed an increased *TUBB* mRNA level in PC-3 cells exposed to s-µg, which is similar to earlier results in endothelial cells [73].

In general, we found a tendency of elevated ECM components at the mRNA level when PC-3 cells were exposed to s-µg on the RPM. The mRNA levels of the *COL1A1*, *COL4A5*, *LAMA3*, *LAMB2*, and *FN1* genes were upregulated or unchanged, with only a few exceptions. In addition, collagen deposition was found in spheroids. A possible explanation for this finding could be that the cells try to increase the ECM to resist the s-µg conditions, because the ECM provides structural support for the cells [74], which they lack after detaching because of the RPM-exposure. Our results concerning the ECM fit the findings of a study of human fetal osteoblasts exposed to s-µg, where the cells showed collagen deposition in spheroids and increased *FN1* and *LAMA1* gene expression [70]. However, the gene expression of *COL4A5*, *FN1*, and *LAMB2* was reduced in s-µg-exposed adult retinal pigment epithelial cells [31]. Thus, regulation of ECM compounds because of µg-exposure must depend on the cell type.

We found an increase in the focal adhesion component *VCL* mRNA. It is plausible that this change occurs because the cells are trying to resist the s-µg conditions and hold onto the ECM, although this does not match the generally unchanged amounts of *TLN1* gene expression in our experiment. In contrast to the results of this study, FTC-133 cells exposed to µg showed no change in *VCL* expression and decreased *TLN* mRNA [75]. Thus, PC-3 cells and FTC-133 cells show a different regulation of FA gene expression and, possibly, synthesis or secretion of proteins.

Finally, we detected a general upregulation of the *CDH1* gene expression. In conjunction with our observations on the secretion of VEGF and NGAL described above, this could also indicate a tendency toward a less-aggressive phenotype, as *CDH1* is a tumor suppressor gene, and inactivation of E-cadherin is involved in the development of prostate cancer [76,77]. E-cadherin was enhanced in papillary thyroid carcinoma cells [78], but decreased in human breast cancer cells [79] exposed to s-µg conditions. Thus, E-cadherin acts depending on the cell type.

### 3.3. Interaction Network of Selected Genes Evaluated by Pathway Analysis

In the pathway analysis various interactions were found, but *VCL*, *FN1*, *mTOR*, *SRC1*, and *VEGFA* are indicated as dominant target genes, as many arrows point to their icons (Figure 6B).

There are interactions between vinculin and fibronectin. *FN1* and *VCL1* were both upregulated in MCS after 3- and 5-day RPM-exposure (Figure 4K and Figure 5B). Similar findings were reported after 24- and 48-h clinostat-exposures of poorly differentiated follicular thyroid cancer cells exhibiting increased expression of vinculin and among other ECM proteins as well as fibronectin in MCS compared with 1 g controls [11]. A recent paper reported that FN1 KO podocytes showed significant downregulated FA molecules (talin, vinculin, and paxillin) and reduced cell spreading, indicating an important role of FN1 in adhesion [80]. FN1 plays a key role in the adaptation of podocytes to mechanical stress. Moreover, this supports the hypothesis that its interaction with vinculin is an adaptive mechanism to protect µg-exposed prostate cancer cells and other cell types.

In addition, there is an interaction between vinculin and MAPK1 (ERK2). Both factors are involved in transmitting and transducing environmental signals to biochemical cascades. The protein kinases are known to regulate the activity of FA proteins [81]. Vinculin also interacts with MAPK3 (ERK1). Macrophages grown on polished surfaces changed from spherical to well-spread cells. These morphological changes were associated with an altered distribution of vinculin [82]. The pFAK, pSrc, pERK1/2 levels were associated with cell shape and a more spread morphology [82].

There is an interesting interaction between FN1 and MAPK3 (ERK2). Treatment of PC-3 cells with 1 μM FN1 resulted in a decrease of activated ERK1/2 [83]. In clear-cell renal cancer, eight differentially expressed genes were identified as biomarkers, including *VEGFA*, *FLT1*, *FN1*, and others [84]. Both fibronectin and VEGF are known to stimulate blood vessel formation. Patient data showed that circulating fibronectin modulates blood vessel formation and tumor growth by modifying the amount of and response to VEGF [85]. In addition, measuring the fibronectin level can serve as a prognostic biomarker for prostate cancer and possibly others.

The *SRC1* mRNA was significantly downregulated in MCS after 3- and 5-day RPM-exposure of prostate cancer cells (Figure 2E), whereas *VCL* was elevated in MCS in our experimental setting (Figure 5B). Moreover, *CDH1* mRNA expression was enhanced in PC-3 MCS after 3 and 5 days on the RPM (Figure 5C). The product of the human SRC gene, *c-Src*, is overexpressed and often activated in many human cancers. The relationship between Src activation and cancer progression appears to be significant [86].

Interestingly, the exposure of PC-3 cells induced a decrease in *SRC1*, which supports the hypothesis that the PC-3 cells showed a more moderate expansion behavior as compared to MCF-7 breast cancer cells. In contrast, in MCF-7 breast cancer spheroids, c-Src was elevated and E-cadherin was reduced [79]. MCS formation could be prevented by inhibition of c-Src and enhanced by blocking of E-cadherin. These results suggest that the balance of proteins that up- or downregulate E-cadherin mediates the tendency of breast cancer cells to form MCS during s-µg-exposure [79].

In addition, there was another detectable interaction between SRC and laminin subunit alpha-3 (LAMA3). *LAMA3* gene expression was upregulated in AD and MCS after a 3-day RPM-exposure of the cells. It also was shown earlier that the recently identified Lm3B11, consisting of laminin α3B (encoded by *LAMA3*), β1, and γ1 chains, stimulated the phosphorylation of Src and Akt more strongly than other laminins in vascular basement membranes [87]. This unique activity of Lm3B11 appears to be favorable to the process of angiogenesis.

SRC has been found to play a crucial role in VEGF-dependent vascular permeability involved in angiogenesis. Chou et al. showed that the two main VEGFRs, kinase insert domain-containing receptor/fetal liver kinase-1 (KDR/Flk-1) and Fms-like tyrosine kinase-1 (Flt-1), interact with SRC. VEGF stimulation elevated SRC activity associated with activated KDR/Flk-1 in endothelial cells [88]. The gene expression of *VEGFA* was downregulated, whereas both VEGFA-receptor genes, *KDR* and *FLT1*, were upregulated in all 5-day RPM samples, which might indicate a counterregulatory effect (Figure 2A–C). In colon cancer cells, SRC induced VEGF, thus enhancing angiogenesis [89,90]. The relationship between Src and VEGF appears to be somewhat reciprocal [86]. VEGF-induced activity is negated with the inhibition of Src activity. Src also appears to provide a link in VEGF signaling of MAPK pathways, contributing to spreading and metastasis [90].

In PC-3 prostate cancer cells, we found a significant downregulation of *VEGFA* mRNA and protein secretion, indicating a lower metastatic potential of the cells when exposed to microgravity. These results are similar to those obtained from low-differentiated follicular thyroid cancer cells in space [47], where we also found a downregulation of both VEGFA gene and protein secretion.

The PI3K/AKT/mTOR signaling pathway plays a key role in cancer metastasis. The activation of a mitogenic pathway involving a feedback mechanism between mTOR and PI3K/ERK1/2 is important for the tumorigenesis of glioblastoma multiforme [91] and may be involved in 3D spheroid formation in s-µg. The MAPK and PI3K/AKT/mTOR pathways regulate cell survival, proliferation, and motility. In addition to their independent signaling programs, the pathways engage in an extensive crosstalk to both positively and negatively regulate each other. Encouragingly, co-inhibition of both pathways has been successful in reducing tumor growth in xenograft cancer models and, importantly, also in genetically engineered mouse models [92,93]. mTOR catalyzes the phosphorylation of multiple targets such as AKT, protein kinase C (PKC), and others, thereby regulating various biological processes such as tumor growth and metastasis. Many mTOR inhibitors have been developed to treat cancer. While some of them have been approved to treat human cancer, more mTOR inhibitors are being evaluated in clinical trials. In addition, current research is designed to optimize the use of VEGF/VEGF receptor inhibitors and mTOR inhibitors for combination or sequential treatment of patients with advanced cancer [94,95].

In short, the s-µg conditions created by the RPM caused differential regulations of the expression of the VEGF, MAPK, and PAM pathway-involved genes *VEGFA*, *FLK1*, *FLT1*, *RAF1*, *SRC1*, *AKT1*, *MTOR*, *MAP2K1*, *ERK1*, and *ERK2*, as well as the genes *ACTB*, *TUBB*, *COL1A1*, *COL4A5*, *LAMA3*, *LAMB2*, *FN1*, *TLN1*, *VCL*, and *CDH1*, involved in signal transduction in the cytoskeleton, ECM, and FAs. Furthermore, a redistribution of F-actin was found in the cells, as well as a deposition of collagen in MCS and decreased secretion of VEGF and NGAL. Thus, s-µg resulted in the regulation of proteins and genes involved in angiogenesis, cell morphology, migration, attachment, ECM, and 3D growth. Possibly, some changes are due to attempts to resist the alterations in µg. Moreover, the PC-3 cells formed spheroids when cultured in s-µg conditions. These 3D constructs might be useful in future studies on the effects of anticancer drugs, as they more closely mimic the cellular environment in actual metastasis, including, for example, the need for diffusion of the drug into the core of the spheroid as opposed to monolayer cell cultures. A future project could be to examine the effects of drugs targeting the proteins regulated in the PC-3 cells when exposed to µg. This was done, for example, with dexamethasone targeting NFκB in breast cancer cells [21], and possible drug investigations are planned for thyroid cancer cells in µg [22]. In addition, it is also important to apply different signaling pathway inhibitors as well as an agonist to investigate the role of µg in prostate cells. Further examination of prostate cancer cells in weightlessness may thus contribute to investigations concerning treatment options and possibly reduce animal experiments in the field of prostate cancer.

## 4. Materials and Methods

### 4.1. Cell Culturing and Microgravity Simulation on the RPM

The human prostatic carcinoma cell line PC-3 was established from a human prostatic adenocarcinoma metastatic to bone, enabling investigations concerning the changes involved in advanced prostate cancer as well as response to treatment [96]. PC-3 cells (ECACC 90112714) were thawed from a frozen aliquot preserved in liquid nitrogen. They were seeded in T75 flasks (Sarstedt, Nümbrecht, Germany) with RPMI 1640 medium (Gibco, Fisher Scientific, Schwerte, Germany) containing 14% fetal bovine serum (FBS) (Sigma-Aldrich) and 1% penicillin/streptomycin (Gibco, Fisher Scientific, Schwerte, Germany). Afterwards, the medium was changed when necessary, using RPMI 1640 medium with 7% FBS and 1% penicillin/streptomycin. When the cells had grown appropriately, they were subcultured in T25 flasks (Sarstedt) and slideflasks (Thermo Scientific, Waltham, MA, USA) and left in 1 g conditions until reaching approximately 60% confluence.

After reaching 60% confluence, all flasks were filled completely with RPMI medium containing 10% FBS and 1% penicillin/streptomycin. Filling the flasks completely and without air bubbles was important to reduce shear stress and disturbance of the cells. Half of the flasks were mounted on the middle frame of the RPM. The other half were placed beside the RPM in 1 g conditions as controls. The RPM used in this experiment (a desktop model from Airbus, Defense and Space, Leiden, the Netherlands) was, along with all flasks, placed in an incubator at 37 °C and 5% CO_2_. Then, the RPM was started, exposing the cells to s-µg conditions for the chosen time periods (3 or 5 days). The RPM simulates µg with a calculated residual acceleration of approximately ~10^−3^ g [97]. The maximal distance to the rotation center was chosen to be 7 cm, and the rotational acceleration was 20°/s^2^. The device was described in detail by Grimm et al. [98].

### 4.2. Sample Collection

Three groups were collected from the RPM experiments: 1 g, AD, and MCS. In addition, aliquots of medium (cell supernatants) were collected for the TRIFMA experiment.

The control cells were collected from the T25 flasks exposed to 1 g conditions. First, several aliquots of medium from the flasks were collected. The rest of the medium was discarded, the cells were washed with 5 mL of phosphate-buffered saline (PBS) and then scraped off the bottom of each flask with a cell scraper, and another 5 mL PBS (Gibco, Fisher Scientific, Schwerte, Germany). The cell suspensions were transferred to tubes and subsequently centrifuged. After discarding the supernatant, the cell pellets were resuspended in PBS and transferred to reaction tubes.

MCS were collected from the T25 flasks from the RPM. First, the flasks were left until the spheroids had all fallen to the bottom. Then, several aliquots of medium were collected, after which the flasks were shaken to allow the MCS to redistribute themselves in the liquid. The medium was transferred to new tubes and centrifuged. The supernatant was discarded and the pellets containing the MCS were resuspended in PBS and transferred to Eppendorf tubes.

AD were collected from the RPM flasks by scraping the cells in PBS, then transferred to Eppendorf tubes.

Following the collection, all Eppendorf tubes were centrifuged, the supernatant was discarded, and pellets were snap-frozen in liquid nitrogen and stored at −80 °C. The supernatant aliquots were frozen and stored at −20 °C. All centrifugation steps were performed at 3000 rpm and 4 °C for 10 min.

### 4.3. RNA Isolation

RNA isolation was done using the RNeasy Mini Kit (Qiagen, Hilden, Germany). The samples were thawed and kept on ice. First, 10 µL of β-mercaptoethanol (BME) was added to 1 mL of lysis buffer (RTL), followed by the addition of 600 µL of this mixture to each sample. The samples were incubated for 2 min and the cells were lysed by applying shear force while passing them through a 20 G needle. Finally, 600 µL of 70% ethanol was added to each sample.

Then 700 µL of each sample was transferred to RNeasy Mini spin columns and 700 µL of Buffer RW1 and 500 µL of Buffer RPE were added. Between these steps the samples were centrifuged for 15 s at 8000× *g*, and flow-through was discarded. Then 500 µL of Buffer RPE was added again, this time followed by centrifugation for 2 min at 8000× *g*, and flow-through was discarded. Finally, the samples were centrifuged for 1 min to dry the membrane and remove any remaining ethanol.

The RNeasy spin columns were placed in new collection tubes, followed by the addition of 35 µL of RNase-free water directly to the membrane. The RNA was eluted from the membrane by centrifugation for 1 min at 8000× *g*.

### 4.4. Quantitative Real-Time Polymerase Chain Reaction

Before performing qPCR, primers were designed using NCBI Primer Blast, which were selective for cDNA by spanning exon–exon junctions and had a melting temperature of around 60 °C. The primers were synthesized by TIB Molbiol (Berlin, Germany). The primers used are listed in Table 1.

The first step was to generate cDNA from the RNA using the high capacity cDNA reverse transcription kit (Applied Biosystems, Foster City, CA, USA). The RNA concentrations of all samples were measured using a NanoDrop. The results were used to calculate the amount of RNA and nuclease-free water needed to obtain 1 µg of RNA in a final volume of 10 µL. The nuclease-free water was pipetted into wells on a 96-well plate, followed by addition of RNA, then 10 µL of master mix was added to each well. This contained RT buffer, dNTP mix, RT random primers, reverse transcriptase, and nuclease-free water. The plate was sealed and centrifuged briefly to eliminate any air bubbles and spin down liquid. The thermal cycler was run using the following program: primer annealing for 10 min at 25 °C reverse transcription for 120 min at 37 °C and enzyme deactivation for 5 min at 85 °C. After this, 50 µL of nuclease-free water was added to each well to obtain a working volume of 70 µL for the next steps.

The qPCR was carried out using FAST SYBR™ Green Master Mix (Applied Biosystems) and the 7500 Fast Real-Time PCR System (Applied Biosystems). First, a master mix was prepared for measurement of all samples in triplicate as well as 3 no-template controls (NTCs). The master mix contained MM Buffer, reverse primer, forward primer, and water. Then, a sub-master mix was prepared for each sample by mixing 42 µL of master mix and 3 µL of cDNA. For each sample, 13 µL of the sub-master mix was pipetted into a 96-well plate in triplicate. NTCs were added to the last 3 wells. The plate was sealed, centrifuged briefly, and stored at 4 °C. When ready to use, the plate was placed in the instrument, and the thermal cycling conditions were set: 95 °C for 20 s, 95 °C for 3 s, and 60 °C for 30 s. The reaction volume was set to 15 µL and the program was run.

In the following analysis the samples were normalized to 18S rRNA. The comparative threshold cycle (ΔΔ*C*_T_) method was utilized to obtain the relative transcription levels; 1 g controls were defined as 100%.

### 4.5. Immunofluorescence

Staining experiments were performed on cells grown in slideflasks that had been cultured on the RPM for 3 or 5 days and their corresponding 1 g controls in order to investigate the change in distribution of F-actin and β-actin.

When the flasks were taken off the RPM, the medium was discarded and the cells were washed three times with PBS and afterwards fixed with 4% paraformaldehyde (PFA; Sigma-Aldrich, St. Louis, MO, USA) in PBS. All slides were stored at 4 °C until ready to use. When ready, the PFA was removed and the slides were washed with PBS, followed by the addition of 0.1% Triton X-100 (Sigma-Aldrich, St. Louis, MO, USA), which was left for 15 min. Then, the slides were removed from the flasks and placed in a dark and moist chamber. Rhodamine–phalloidin (for F-actin; Sigma, P1951, diluted 1:250) or primary antibody (for β-actin; Sigma, A5316, diluted 1:1000) diluted in 0.5% bovine serum albumin (BSA) (Merck, Darmstadt, Germany) in PBS was pipetted onto each slide. For β-actin, the incubation was left overnight, and the next day secondary antibody (anti-mouse IgG Fab2 Alexa Fluor^®^ 488 Molecular Probes 2 mg/mL #4408S, diluted 1:500) diluted in PBS was added. The secondary antibody was left on the slides for a minimum of 4 h.

Finally, the slides were prepared with Fluoroshield™ mounting medium containing 4′,6′-diamidino-2-phenylindole (DAPI) (Sigma-Aldrich, St. Louis, MO, USA). The slides were sealed by putting a coverslip on top and stored in a dark box at 4 °C. Between steps, the slides were washed with cold PBS.

### 4.6. Histochemical Staining

Histological staining was performed on 3- and 5-day 1 g controls and RPM slides using hematoxylin-eosin (HE) and Sirius red (SR) stains as described earlier [99,100].

The histological staining was done on cells on slides and on MCS on separate slides. After culturing the cells on the RPM for 3 or 5 days, the MCS were collected from the slides with a pipette, washed three times in PBS, and then fixed in 4% PFA. Then they were embedded in paraffin, followed by sectioning into 3 µm sections with a microtome. For 1 g and RPM AD samples, the medium was discarded and the slides were washed three times with PBS, and afterwards 4% PFA was applied. The slides and sectioned spheroids were stained with HE or SR. HE staining was used to evaluate the cell morphology, while SR staining was used to investigate the collagen content.

### 4.7. Microscopy

All flasks containing viable cells were examined throughout growth as well as before and after RPM-exposure with a Leica phase contract microscope. Pictures were taken using a Canon EOS 550D camera. All cells stained by immunofluorescence were examined with a Leica DFC310 FX fluorescence microscope. Pictures were taken at 20×, 40×, and 100× magnification using the LAS V3.7 software. The pictures were not taken using the same settings, as the signal intensities were not quantified. The pictures were instead used to examine the locations of the proteins. Immersion oil (Sigma-Aldrich, St. Louis, MO, USA) was applied to the slides when using the higher resolution.

### 4.8. Time-Resolved Immunofluorometric Assay

TRIFMA was performed as described earlier [101,102]. In brief, microtiter 96-well plates were coated overnight at 4 °C with primary antibodies dissolved in PBS. They were specific for either NGAL (1 µg/mL) or VEGF (2 µg/mL). All wells were then incubated with blocking buffer (1% Tween-20 in PBS) for 2 h.

The samples were diluted 1:2 in assay buffer to a total of 100 µL and added to the plates. The assay buffer used was PBS supplemented with 0.05% Tween-20. In addition, recombinant standards (VEGF and NGAL) were added to the plate (100 µL per well). Negative control samples, containing 100 µL of assay buffer without added supernatant, were also added to the plates. All samples were added to the plates in duplicate to increase the precision of the results.

The Fluorescence was measured using a time-resolved fluorometer (PerkinElmer, Waltham, MA, USA). The concentrations of VEGF and NGAL were determined using a 4-parameter standard curve fit implemented in WorkOut 2.5 data analysis software (PerkinElmer, Waltham, MA, USA).

### 4.9. Pathway Analysis

To investigate the mutual regulation of genes and visualize localization and interactions between proteins, we entered relevant UniProtKB entry numbers in the Pathway Studio plus software (Elsevier Research Solutions, Amsterdam, The Netherlands). Graphs were generated for gene expression and protein regulation and binding. The method was described previously [103].

### 4.10. Statistical Analysis

All statistical evaluations were performed using IBM SPSS Statistics 23 (IBM Deutschland GmbH, Ehningen, Germany). The Mann–Whitney U-test was utilized to evaluate the statistical significance of the changes in expression levels following RPM exposure, thus comparing 1 g control to AD and MCS. A significance level of 0.05 was used. The standard deviation was calculated and presented together with the mean values as percentages in bar plots.

## 5. Conclusions

Prostate cancer cells (PC-3 cell line) exposed to s-µg conditions generated by an RPM grew as adherent cells and in the form of 3D MCS. The RPM-exposed cells revealed alterations in the cytoskeleton, as well as gene expression changes of cytoskeletal and FA factors and ECM components. The VEGF, MAPK, and PAM signaling pathways are involved in 3D formation of prostate cancer cells. The majority of the factors are upregulated, indicating their impact on growth and progression of these cells.

Overall, our study suggests that these pathways drive the regulation of metastasis, survival, and angiogenesis of prostate cancer cells.

## Figures and Tables

**Table 1:** List of primer sequences used in quantitative PCR. All sequences are listed in the 5′–3′ direction.

Factor	Primer Name	Sequence 5′–3′
18S-rRNA	18s-F	GGAGCCTGCGGCTTAATTT
	18s-R	CAACTAAGAACGGCCATGCA
Actin-beta (***ACTB***)	ACTB-F	TGCCGACAGGATGCAGAAG
	ACTB-R	GCCGATCCACACGGAGTACT
RAC-alpha Serine/threonine-protein kinase (***AKT1***)	Akt1-F	CTTCTATGGCGCTGAGATTGTG
	Akt1-R	CAGCATGAGGTTCTCCAGCT
Collagen 1 alpha 1 (***COL1A1***)	COL1A1-F	ACGAAGACATCCCACCAATCAC
	COL1A1-R	CGTTGTCGCAGACGCAGAT
Collagen 4 alpha 5 (***COL4A5***)	COL4A5-F	GGTACCTGTAACTACTATGCCAACTCCTA
	COL4A5-R	CGGCTAATTCGTGTCCTCAAG
E-cadherin (***CDH1***)	CDH1-F	GCTGGACCGAGAGAGTTTCC
	CDH1-R	CAGCTGTTGCTGTTGTGCTT
Extracellular signal-regulated kinase 1 (***ERK1***)	ERK1-F	ACCTGCGACCTTAAGATTTGTGA
	ERK1-R	AGCCACATACTCCGTCAGGAA
Extracellular signal-regulated kinase 2 (***ERK2***)	ERK2-F	TTCCAACCTGCTGCTCAACA
	ERK2-R	TCTGTCAGGAACCCTGTGTGAT
Focal adhesion kinase 1 (Protein-tyrosin kinase 2) (FAK1 (***PTK2***))	FAK1-F	TGTGGGTAAACCAGATCCTGC
	FAK1-R	CTGAAGCTTGACACCCTCGT
Fibronectin (***FN1***)	FN1-F	TGAGGAGCATGGTTTTAGGAGAA
	FN1-R	TCCTCATTTACATTCGGCGTATAC
Laminin alpha 3 (***LAMA3***)	LAMA3-F	AAAGCAAGAAGTCAGTCCAGC
	LAMA3-R	TCCCATGAAGACCATCTCGG
Laminin β2 (***LAMB2***)	LAMB2-F	TGCTCATGGTCAATGCTAATCTG
	LAMB2-R	TCTATCAATCCTCTTCCTTGGACAA
Mitogen-activated protein kinase kinase 1 (***MAP2K1***)	MAP2K1-F	CGTTACCCGGGTCCAAAATG
	MAP2K1-R	TCCAAGTTGGTCTCCGCA
Mechanistic target of rapamycin kinase (***MTOR***)	MTOR-F	ATCTTGGCCATAGCTAGCCTC
	MTOR-R	ACAACTGGGTCATTGGAGGG
Neutrophil gelatinase-associated lipocalin (NGAL, ***LCN2***)	LCN2-F	AGGGAGTACTTCAAGATCACCCTCTA
	LCN2-R	AGAGATTTGGAGAAGCGGATGA
Raf-1 proto-oncogene, serine/threonine kinase (***RAF1***)	RAF1-F	GGGAGCTTGGAAGACGATCAG
	RAF1-R	ACACGGATAGTGTTGCTTGTC
Rapamycin-insensitive companion of MTOR (***RICTOR***)	RICTOR-F	GGAAGCCTGTTGATGGTGAT
	RICTOR-R	GGCAGCCTGTTTTATGGTGT
Steroid receptor coactivator-1 (***SRC1***)	SRC1-F	CCACCTTTGTGGCCCTCTAT
	SRC1-R	CCTCTGTGTTGTTGACAATCTGG
Talin-1 (***TLN1***)	TLN1-F	GATGGCTATTACTCAGTACAGACAACTGA
	TLN1-R	CATAGTAGACTCCTCATCTCCTTCCA
Tubulin-beta (***TUBB***)	TUBB-F	CTGGACCGCATCTCTGTGTACTAC
	TUBB-R	GACCTGAGCGAACAGAGTCCAT
Vascular endothelial growth factor A (***VEGFA***)	VEGFA-F	GCGCTGATAGACATCCATGAAC
	VEGFA-R	CTACCTCCACCATGCCAAGTG
Vascular endothelial growth factor receptor 1 (***FLT1***)	FLT1-F	CCCTCGCCGGAAGTTGTAT
	FLT1-R	GATAATTAACGAGTAGCCACGAGTCAA
Vascular endothelial growth factor receptor 2 (***FLK1***)	FLK1-F	TCTTCTGGCTACTTCTTGTCATCATC
	FLK1-R	GATGGACAAGTAGCCTGTCTTCAGT
Vinculin (***VCL***)	VCL-F	GTCTCGGCTGCTCGTATCTT
	VCL-R	GTCCACCAGCCCTGTCATTT

## Abbreviations

AD	Adherent cell(s)
BME	β-mercaptoethanol
BSA	Bovine serum albumin
DAPI	4′,6-diamidino-2-phenylindole
ECM	Extracellular matrix
EMT	Epithelial–mesenchymal transition
ERK	Extracellular signal regulated kinase
FA	Focal adhesion
FAK	Focal adhesion kinase
FLK1	Fms-related tyrosine kinase 1
FLT1	Fetal liver kinase 1
FBS	Fetal bovine albumin
HE	Hematoxylin–eosin
HRP	Horseradish peroxidase
IF	Immunofluorescence
MAPK	Mitogen-activated protein kinase
MCS	Multicellular spheroid(s)
MEK1	Mitogen-activated protein kinase kinase 1
mTOR	Mammalian target of rapamycin
mTORC2	mTOR complex 2
µg	Microgravity
NGAL (LCN2)	Neutrophil gelatinase-associated lipocalin (lipocalin 2)
NTC	No-template control
1 g	Normal gravity
PAM	PI3K/AKT/mTOR
PBS	Phosphate-buffered saline
PBST	Phosphate-buffered saline with Tween
PFA	Paraformaldehyde
PI3K	Phosphoinositide 3-kinase
PKB	Protein kinase B
qPCR	Quantitative real-time polymerase chain reaction
RAF	Rapidly accelerated fibrosarcoma
RICTOR	Rapamycin-insensitive companion of mTOR
RIPA	Radioimmunoprecipitation assay
RPM	Random positioning machine
rpm	Rounds per minute
RWV	Rotating wall vessel
SR	Sirius red
SRC (Src)	Steroid receptor coactivator (proto-oncogene, non-receptor tyrosine kinase)
S-µg	Simulated microgravity
TBST	Tris-buffered saline with Tween
VEGF	Vascular endothelial growth factor
VEGFR	Vascular endothelial growth factor receptor
WHO	World Health Organization
TRIFMA	Time-resolved immunofluorometric assay
2D	Two-dimensional
3D	Three-dimensional
